# Further evidence of the involvement of the Wnt signaling pathway in Dupuytren’s disease

**DOI:** 10.1007/s12079-015-0312-8

**Published:** 2015-12-03

**Authors:** Evert-Jan P. M. ten Dam, Marike M. van Beuge, Ruud A. Bank, Paul M. N. Werker

**Affiliations:** 1 Department of Pathology & Medical Biology, University of Groningen and University Medical Center Groningen, EA11, P.O. Box 30 001, 9700 RB, Groningen, The Netherlands; 2 Department of Plastic Surgery, University of Groningen and University Medical Center Groningen, BB81, P.O. Box 30 001, 9700 RB, Groningen, The Netherlands

**Keywords:** β-catenin, Dupuytren’s disease, Fibrosis, Wnt signaling

## Abstract

Genetic background plays an important role in the development of Dupuytren’s disease. A genome-wide association study (GWAS) showed that nine loci are associated with the disease, six of which contain genes that are involved in Wnt signaling (*WNT2*, *WNT4*, *WNT7B*, *RSPO2*, *SFRP4*, *SULF1*). To obtain insight in the role of these genes, we performed expression studies on affected and unaffected patient’s tissues. Surgically obtained nodules and cords from eight Dupuytren’s patients were compared to patient-matched control tissue (unaffected transverse palmar fascia). The Wnt-related genes found in the GWAS, the classical Wnt-downstream protein β-catenin, as well as (myo)fibroblast markers were analyzed using real-time qPCR and immunohistochemical stainings for mRNA levels and protein levels, respectively. The collagen-coding genes *COL1A1* and *COL3A1* were highly upregulated on mRNA level, both in cords and nodules. Three Wnt-related genes were found to be differently regulated compared to control tissue: *WNT2* was downregulated in nodules, *WNT7B* was upregulated in nodules, and *SFRP4* was upregulated in nodules and cords. Immunohistochemistry revealed significantly less staining of Wnt2 in cords, but significantly more staining for Wnt7b in nodules. There was significantly more staining of α-SMA in nodules and cord and β-catenin in nodules than in control tissue. We found differences in expression, both at mRNA and protein level, in several Wnt-related genes found earlier to be associated with Dupuytren’s disease. Of these, Wnt7b was upregulated and found in close association with both α-SMA and β-catenin expressing cells, making it a candidate pro-fibrotic mediator in Dupuytren’s disease.

**Electronic supplementary material:**

The online version of this article (doi:10.1007/s12079-015-0312-8) contains supplementary material, which is available to authorized users.

## Introduction

Dupuytren’s disease is a benign fibroproliferative disorder of the hand, which causes the formation of nodules and cords in the palm and fingers. It may eventually lead to the inability to fully extend the fingers. The prevalence varies from 1 % to 32 % in Western countries (Lanting et al. 2013). The disease is more common in people of European ancestry, in older persons and in males (Gudmundsson et al. 2000; Hindocha et al. 2009). The main treatment option has until recently been open surgery, but use of less invasive methods, such as percutaneous needle fasciotomy and collagenase injections in the cords, is becoming more popular (van Rijssen et al. 2012; Hurst et al. 2009). However, there is no definitive cure and recurrences are frequent (van Rijssen et al. 2012).

Pathophysiologically, both contraction and matrix deposition caused by uncontrolled myofibroblast activity in and around the palmar fascia of the hand are key features (Tomasek et al. 1987). The development of myofibroblasts in general depends on a number of different environmental cues, including tension in the matrix and exposure to a variety of different mediators, such as transforming growth factor-β1 (Hinz 2007). Myofibroblasts have been suggested to make up the majority of nodular cells, with cords being less cellular and more tendon-like (Verjee et al. 2009).

Several causes have been proposed for Dupuytren’s disease, and a genetic component is one of them. Concerning this genetic predisposition, in a genome-wide association study (GWAS), nine chromosomal loci were found to be associated with susceptibility to Dupuytren’s disease (Dolmans et al. 2011). Six of these loci contain genes involved in the Wnt signaling pathway. The canonical pathway of Wnt signaling is the most extensively studied and has been shown to promote cell proliferation and survival via β-catenin (Moon et al. 2004; Rao and Kuhl 2010). Alternatively, Wnt proteins may signal via the non-canonical Wnt pathway, defined as all Wnt signaling activities that operate independently of β-catenin.

**Fig. 1:**
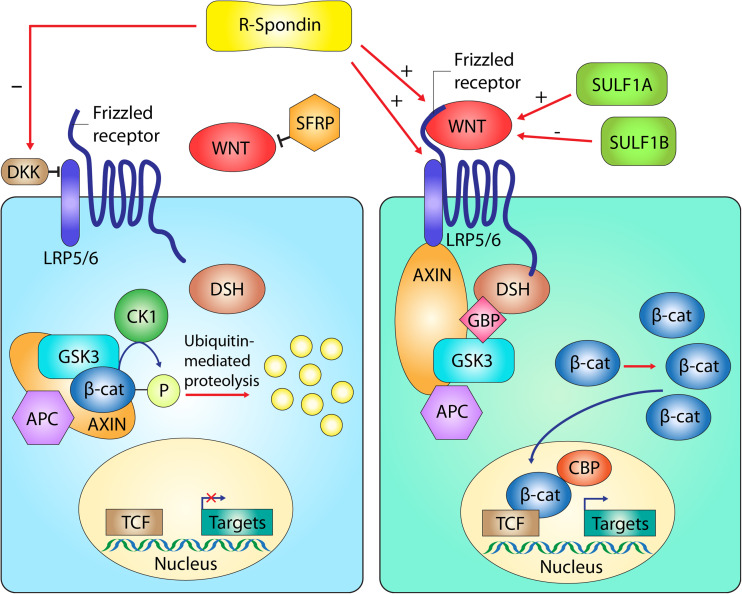
The tested Wnt-related genes and proteins and their functions in the canonical Wnt signaling pathway are shown here. On the left, the Wnt pathway is inhibited: β-catenin (β-cat) is degraded in the abcense of a Wnt protein. On the right, the Wnt pathway is activated: a translocation of β-catenin to the nucleus when a Wnt protein binds to the Frizzled receptor

The Wnt-related genes that have been identified in the GWAS are *WNT2*, *WNT4*, *WNT7B*, *RSPO2*, *SFRP4* and *SULF1* (Fig. 1). Of these, three Wnt proteins exert their effect through binding to Frizzled receptors, causing a nuclear translocation of β-catenin via the canonical pathway. They may also activate the non-canonical pathway via Frizzled and other receptors. Secreted Frizzled-related proteins (Sfrp) bind to Wnt proteins, thereby inhibiting normal Wnt-Frizzled interactions. R-Spondin activates the pathway by interacting with Frizzled receptors, Lrp5/6 and Dkk proteins (Rao and Kuhl 2010). Sulf1 is a member of the sulfatase gene family that also interacts with canonical Wnt signaling, although the mechanism is unclear: both activation and inhibition of Wnt signaling have been described (Sahota and Dhoot 2009).

Although the association between the Wnt pathway and Dupuytren’s disease, as described by Dolmans et al., has been confirmed in three independent association studies (Dolmans et al. 2011; Shih et al. 2012; Anderson et al. 2014). the expression/involvement of Wnt pathway members in diseased Dupuytren’s tissue is unclear. An increased protein expression and nuclear translocation of β-catenin has repeatedly been reported (Varallo et al. 2003; Howard et al. 2003; Montgomery and Folpe 2005; O’Gorman et al. 2006; Degreef et al. 2009; Vi et al. 2009), but reports on mRNA expression and protein localization data on the Wnt pathway are rare and seem to be contradictory (O’Gorman et al. 2006; Degreef et al. 2009). In this study we have investigated the expression and protein localization of the six genes identified in the GWAS in affected nodules and cords of eight Dupuytren’s patients, and used unaffected fascia of the same donors as control tissue. By doing so, we were able to investigate the presence of mRNA levels of several Wnt-related genes and their corresponding proteins in affected and non-affected tissues. Collagen type I and III and alfa-smooth muscle actin (α-SMA) were measured to obtain an impression of the diseased state of the cords and nodules.

## Material and methods

### Primary tissues

Dupuytren’s nodules, cords and unaffected transverse ligaments of the palmar aponeurosis (TLPA) were obtained from Dupuytren’s patients with primary disease that underwent limited fasciectomy or dermofasciectomy by plastic surgeons in the University Medical Center Groningen. Nodules and cords were considered affected, and TLPA was considered unaffected (control). All affected and control tissues were patient-matched.

### Quantitative RT-PCR

Up to 30 mg of tissue per sample was cut into small pieces and disrupted using an ultra-turrax. RNA extraction was performed using the RNeasy Fibrous Tissue Mini Kit (Qiagen, Hilden, Germany), according to the manufacturer’s instructions. RNA was quantified using a NanoDrop-1000 spectrophotometer (NanoDrop Technologies, Wilmington, DE). Complementary DNA was synthesized from RNA using the RevertAid First Strand cDNA Synthesis Kit (Thermo Scientific, Rockford, IL). Primers were ordered from Sigma-Aldrich (Zwijndrecht, The Netherlands) (sequences: Table 1). Samples were analyzed in triplo (Wnt-related) or duplo (others) and pipetted onto MicroAmp Optical 384-Well Reaction Plates with Barcode (Applied Biosystems, Foster City, CA). Plates were run on the ViiA 7 Real-Time PCR system (Applied Biosystems). The relative amount of product was calculated using the ΔΔCt method, normalizing for the averaged expression of the household genes *GAPDH* and *YWHAZ* and related to the control tissue.

**Table 1:** Primers for the genes used for qPCR

**Gene**	**Forward**	**Reverse**
*WNT2*	atgggagcatcagtatgcaa	tccctgaatgtcatcttttgg
*WNT4*	gcagagccctcatgaacct	cCacccgcatgtgtgtcag
*WNT7B*	cacagaaactttcgcaagtgg	ggctaggccaggaatcttgtt
*RSPO2*	cccacgtgctaaccaagc	catctctccgccacgaac
*SFRP4*	gcctgaagccatcgtcac	ccatcatgtctggtgtgatgt
*SULF1*	gcgttcatcatacatcataccttt	tcatgccaagaaaaccaaaa
*ACTA2*	ctgttccagccatccttcat	tcatgatgctgttgtaggtggt
*COL1A1*	gcctcaaggtattgctggac	accttgtttgccaggttcac
*COL3A1*	ctggaccccagggtcttc	catctgatccagggtttcca
*GAPDH*	agccacatcgctcagacac	gcccaatacgaccaaatcc
*YWHAZ*	gatccccaatgcttcacaag	tgcttgttgtgactgatcgac

### Immunohistochemistry

Frozen tissue on Tissue-Tek (Sakura, Zoeterwoude, The Netherlands) was cut into 5–10 μm coupes and put onto microscope slides (Starfrost**,** Waldemar Knittel GmbH, Brunschweig, Germany). Sections were fixed for 10 min with acetone (stainings for Wnt2, Wnt7b, Sfrp4, α-SMA), or 4 % paraformaldehyde in PBS with permeabilization using 0.1 % Triton X (stainings for Wnt4, Rspo2, Sulf1, β-catenin, Collagen-I, Collagen-III). The sections were incubated with a primary antibody for 60 min at room temperature, with 1 % bovine serum albumin (antibody information: see Table 2). Endogenous peroxidases were blocked using 0.1 % H_2_O_2_ during 15 min. For all biotinylated secondary antibodies, avidin and biotin were blocked for 15 min using the Biotin Blocking System (Dako, Glosstrup, Denmark). Secondary antibodies (Dako and Southern Biotech, Birmingham, AL) diluted 1:100 with 2 % human serum, were incubated during 30 min. The stainings were visualized using 3-amino-9-ethylcarbazole (AEC; Sigma-Aldrich) or Vector Red (Vector Laboratories, Burlingame, CA). Hematoxylin (Merck, Darmstadt, Germany) was used as counterstaining and all slides were mounted in Kaiser’s glycerin-gelatin (Merck).

**Table 2:** Primary antibodies used for immunohistochemical stainings

**Antibody**	**Manufacturer**	**Product Code**	**Dilution**
Wnt2	R&D, Minneapolis, MN	AF3464	1:50
Wnt4	Sigma-Aldrich, Zwijndrecht, The Netherlands	HPA011397	1:50
Wnt7b	Sigma-Aldrich, Zwijndrecht, The Netherlands	SAB2104506	1:200
R-Spondin2	Santa Cruz Biotechnology, Santa Cruz, CA	SC-74,883	1:100
Sfrp4	Sigma-Aldrich, Zwijndrecht, The Netherlands	HPA009712	1:25
Sulf1	Santa Cruz Biotechnology, Santa Cruz, CA	SC-98,325	1:100
β-catenin	BD Transduction Laboratories, San Diego, CA	610,153	1:100
α-SMA	Dako, Glosstrup, Denmark	M0851	1:100
Collagen-I	Abcam, Cambridge, MA	6308	1:5000
Collagen-III	Abcam, Cambridge, MA	6310	1:5000

### Quantification of stainings

Stainings were analyzed using a Leica DM 2000 microscope (Leica, Wetzlar, Germany). For quantification, three to five representative photomicrographs (40× magnification) were taken per tissue section, and analyzed using Nuance 3.0 software (PerkinElmer, Waltham, MA), allowing detection of a specific signal without interfering background noise. For each specific staining, stained areas were quantified as μm^2^/high power field.

### Statistics

Statistical analysis was performed using GraphPad Prism version 5 (GraphPad Software, La Jolla, CA). A Kruskal-Wallis test was used to determine whether there were differences in ranks between the three groups. A post-hoc Dunn’s Multiple Comparisons test compared controls with cords and controls with nodules. *P* values <0.05 were considered to be statistically significant. Graphs show individual sample values, accompanied by a mean with SEM whisker plot per group. Significances are shown as * (*P* < 0.05); ** (*P* < 0.01) and *** (*P* < 0.001).

## Results

### Characterization of tissue: collagen and α-smooth muscle actin

Tissues were characterized by real-time qPCR and immunohistochemical stainings on nodules, cords and control tissue in terms of expression of *ACTA2*/α-SMA, *COL1A1*/collagen type I and *COL3A1*/collagen type III. We did not find significant differences in mRNA expression of *ACTA2* between affected tissues and control, but observed a high upregulation of both *COL1A1* and *COL3A1* in both cords and nodules (Supplemental Fig. 1a). At protein level we found significantly more α-SMA, the main marker for myofibroblasts, in cord and nodules than in control tissue (Supplemental Fig. 1b). These stainings clearly showed a pattern of different zones of high intensity within the affected tissues. Both collagens were highly upregulated in cords at protein level, while collagen type III only showed a significant upregulation in nodules (Supplemental Fig. 1c-e).

### MRNA expression of Wnt-related genes

**Fig. 2:**
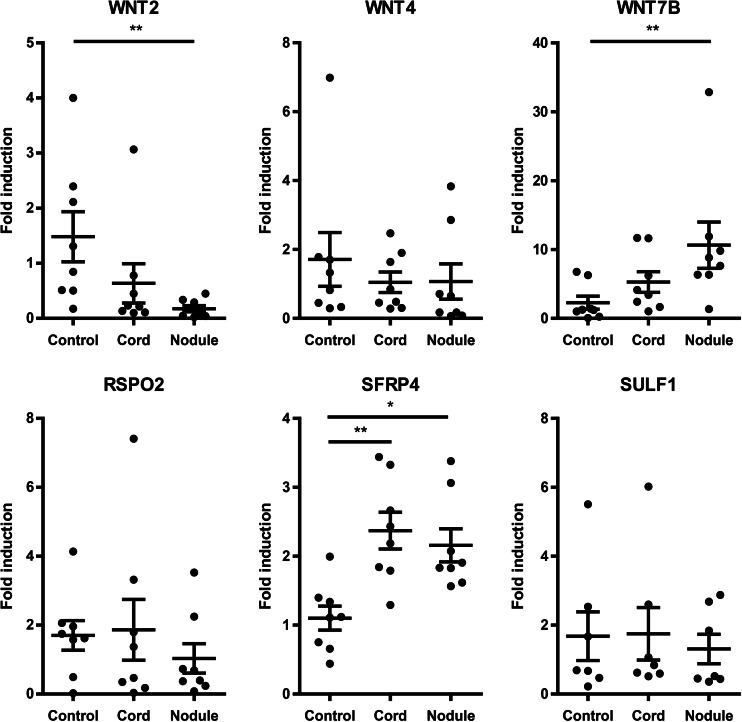
mRNA levels of WNT-related genes in Dupuytren’s disease tissue (cord, nodule) as compared to control tissue (unaffected transverse ligaments of the palmar aponeurosis). Upper panel: *WNT2*, *WNT4*, *WNT7B*; lower panel: *RSPO2*, *SFRP4* and *SULF1*. * *P* < 0.05, ** *P* < 0.01 by Kruskal-Wallis test, followed by post-hoc Dunn’s Multiple Comparisons test

The six Wnt-related genes that were found to be associated with Dupuytren’s disease in the GWAS (Dolmans et al. 2011) were analyzed in eight (*SULF1*: seven) affected cords and nodules and in unaffected TLPA by performing real-time qPCR. Significant expression differences in three of these genes were found in affected tissue compared to control tissue: a nine-fold downregulation of *WNT2* in nodules (*P* < 0.01), a five-fold upregulation of *WNT7B* in nodules (*P* < 0.01), and a two-fold upregulation of *SFRP4* in cords as well as nodules (*p* < 0.01). (Fig. 2) We did not find any significant differences in expression of *WNT4*, *RSPO2* and *SULF1*.

### Immunohistochemistry

**Fig. 3:**
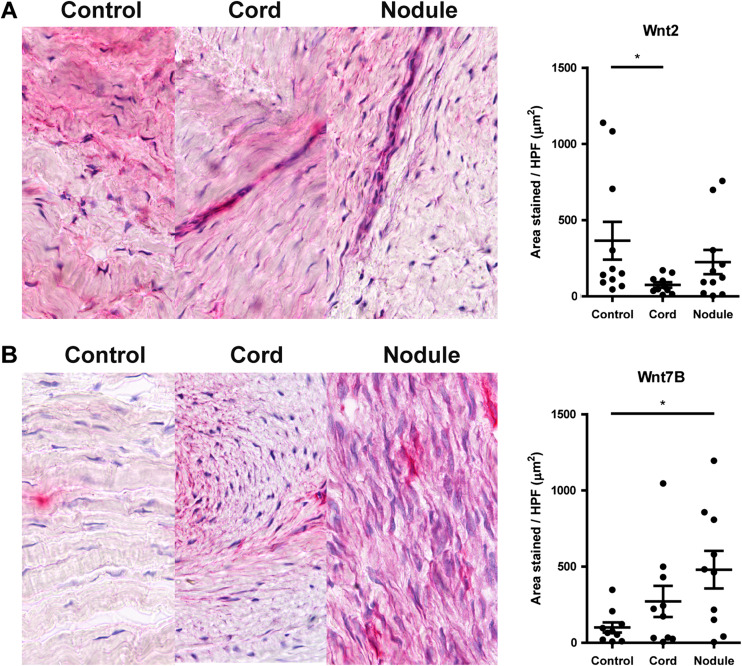
Representative pictures and quantification of staining of selected Wnt-proteins in Dupuytren’s disease tissue (cords, nodule) as compared to control tissue (unaffected transverse ligaments of the palmar aponeurosis). **a** Wnt2. **b** Wnt7b. * *P* < 0.05 by Kruskal-Wallis test, followed by post-hoc Dunn’s Multiple Comparisons test

**Fig. 4:**
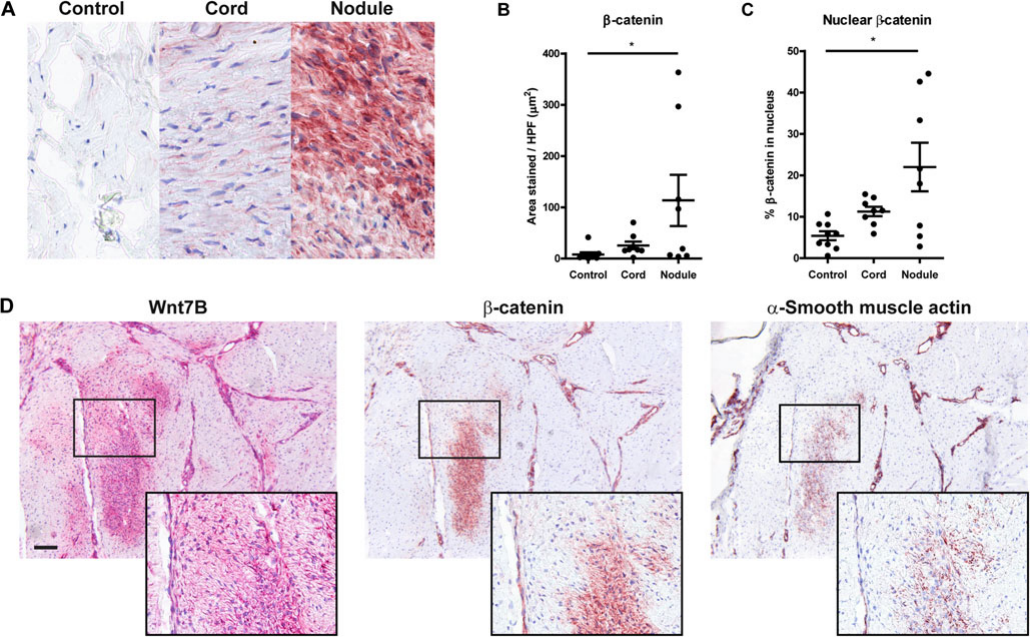
Representative pictures and quantification of staining of β-catenin in Dupuytren’s disease tissue (cord, nodule) as compared to control tissue (unaffected transverse ligaments of the palmar aponeurosis). **a** photomicrographs. **b** quantification of staining for β-catenin. * *P* < 0.05 by Kruskal-Wallis test, followed by post-hoc Dunn’s Multiple Comparisons test. **c** percentage of nuclear localization measured as colocalization of β-catenin positive signal with hematoxylin signal. * *P* < 0.05 by Kruskal-Wallis test, followed by post-hoc Dunn’s Multiple Comparisons test. **d**) Staining for Wnt7b, β-catenin and α-smooth muscle actin (scale bar represents 100 μm) in serial sections of a Dupuytren’s nodule

Significantly less staining of the Wnt2 protein was observed in cords than in controls (*P* < 0.05). Also, we found more Wnt7b staining (*P* < 0.05) in nodules than in controls (Fig. 3). Both results are in agreement with the qPCR results (Fig. 2). There were no significant differences in staining for Wnt4, Rspo2, Sfrp4 and Sulf1 (data not shown). β-catenin, which is the common downstream target of the canonical Wnt pathway, showed significantly more staining in nodules than in controls (*P* < 0.05) (Fig. 4a,b). A larger percentage of this staining was associated with the nucleus compared to controls (Fig. 4c). Finally, using staining of serial sections, we found that stainings for Wnt7b and β-catenin clustered in or around the zones with a positive α-SMA staining (Fig. 4d).

## Discussion

We examined the expression of the Wnt-related genes found to be associated with Dupuytren’s disease in the GWAS by Dolmans et al. (2011). as well as the corresponding proteins and the Wnt downstream target β-catenin. We found that several Wnt-related genes were differentially regulated in affected tissue compared to control tissue. Specifically, we report upregulation of *WNT7B* and *SFRP4* and downregulation of *WNT2*. We also found a clear increase in expression of Wnt-downstream protein β-catenin in Dupuytren’s nodules, especially in nuclei, strongly suggesting an overall activation of the canonical Wnt signaling pathway in Dupuytren’s disease.

Dolmans et al. (2011) found single nucleotide polymorphisms in six areas where several Wnt genes are coded to be associated with Dupuytren’s. They concluded that these genes could be relevant in the occurrence and development of the disease. In accordance with this, we show three of these genes to be differentially expressed between normal and affected tissues within patients, extending the findings of the GWAS study. Not all Wnt-related genes found in the GWAS showed an altered regulation in our study. Our limited study size, as well as the different study design, since we used internal instead of external controls, might be responsible for this. A previous study showed that differences between affected fibroblasts and fibroblasts from unaffected fascia in Dupuytren’s patients are smaller than those between palmar fascia fibroblasts from healthy persons and affected fibroblasts from Dupuytren’s patients (Satish et al. 2012). In line with these findings, we did indeed find less differences, however, these can all be ascribed to the disease process, since inter-patient variation does not play a role in our study set-up. Besides, TLPA may seem unaffected during surgery, but contain microscopical spurs of disease that influence the outcome of PCR and immunohistochemical examinations. Expression profiles of *ACTA2*/α-SMA, *COL1A1*/collagen type I and *COL3A1*/collagen type III on mRNA and/or protein level confirmed that the affected tissues were fibrotic, as compared to the control TLPA. These data contribute to the validation of our findings regarding Wnt signaling and β-catenin in Dupuytren’s tissues.

Of the 19 Wnt-ligand genes in the human genome (Clevers and Nusse 2012) we studied three: *WNT2*, *WNT4* and *WNT7B*. Next to these Wnt ligands, we examined three genes that influence Wnt signaling: *SFRP4*, *RSPO2* and *SULF1*. The Wnt-related genes that we analyzed have different functions in the Wnt signaling pathway. We found the *WNT2* gene to be lower expressed in Dupuyten’s nodules, and the Wnt2 protein to have less staining in cords compared to controls. In view of the general profibrotic role ascribed to the Wnt pathway this is a remarkable finding. Wnt2 has been specifically linked to fibrosis before, for instance by Bayle et al., who found an increased mRNA expression of *WNT2* in a mouse model of skin fibrosis (Bayle et al. 2008). Possibly, a negative feedback mechanism in which the *WNT2* gene is being suppressed as a reaction to excessive activation of the Wnt signaling pathway, plays a role in Dupuytren’s disease. In this context, it is of interest to note that *WNT2* was also found to be a susceptibility locus in the related Peyronie’s disease, in which the penis is affected (Dolmans et al. 2012). However, it is not known whether in this disease *WNT2* is up- or downregulated. Further study is needed to prove a similar downregulation in Peyronie’s disease.

In contrast to the lower expression of the *WNT2* gene and protein in Dupuytren’s tissue, we found a significantly higher expression of the *WNT7B* gene in nodules compared to control tissue, and more Wnt7b positive cells in nodules. This gene has been reported to play a role in several pulmonary conditions. A higher expression of *WNT7B* was found in lung tissue from patients with idiopathic pulmonary fibrosis, on mRNA (Konigshoff et al. 2008) and protein level. The increased immunostaining of the Wnt7b protein was found especially in regions with fibrotic changes (Meuten et al. 2012). which is in accordance with our findings in nodule, where Wnt7b staining was found in α-SMA positive areas. Overall, these and our findings seem to pinpoint *WNT7B* as an important effector molecule in fibrosis. If so, a higher expression of *WNT7B* in affected tissue of Dupuytren’s patients seems a likely candidate to be connected with the disease process, either as a cause or consequence.

We found a significantly (two-fold) higher expression of the *SFRP4* gene in cords and nodules, but no differences in protein staining. *SFRP4* codes for one of the secreted Frizzled-related proteins, a group of Wnt-binding proteins that inhibit interactions between Wnt and Wnt receptors (Clevers and Nusse 2012). but it has also been reported to activate Wnt signaling (Mahdi et al. 2012). Shih et al. found a higher mRNA expression of *SFRP4* in Dupuytren’s tissues (Shih et al. 2012). In humans with systemic sclerosis, an upregulation of *SFRP4* on mRNA and protein level was found in fibrotic skin (Bayle et al. 2008). On the other hand, Sfrp4 administration in rats reduces fibrosis after ischemic injury to the heart, and a therapeutical effect of recombinant Sfrp4 on renal fibrosis in mice has been described (Matsushima et al. 2010; Surendran et al. 2005). Our findings showed a higher transcriptional expression of *SFRP4*, but – in the absence of a change in protein levels – the effect of this remains unclear.

Several studies have reported increased β-catenin in Dupuytren’s disease and discussed a dysregulation of the Wnt signaling pathway as an upstream regulator of β-catenin (Varallo et al. 2003; Montgomery and Folpe 2005; O’Gorman et al. 2006; Degreef et al. 2009). Varallo et al. described higher levels of β-catenin in diseased palmar fascia as compared to patient-matched normal fascia, with both cytoplasmic and nuclear staining. They suggested that Wnt signaling might contribute to these findings, but a role of TGF-β and the extracellular matrix was also suggested (Varallo et al. 2003). O’Gorman et al. found that changes in Wnt expression are unlikely to be the cause of dysregulation of β-catenin expression in Dupuytren’s disease. They reported that overall Wnt expression, with the exception of *WNT10B*, was unchanged between normal and disease palmar fascia on mRNA level, but they did not examine protein expression (O’Gorman et al. 2006). Degreef et al. found evident staining of β-catenin in involutional zones of Dupuytren’s nodules, without comparing it to control tissue. They found no differences between nodules of patients with recurrent and non-recurrent disease (Degreef et al. 2009). We found significantly more staining of β-catenin in nodules than in control tissue, and the relative amount of staining associated with nuclei was much higher than in controls, confirming activation of the Wnt signaling cascade. In view of the high expression of Wnt7b in the same region as nuclear β-catenin, this protein might be among the Wnt proteins causing the activation of the Wnt pathway in this tissue.

Based on our results, the Wnt signaling pathway is (over)activated in Dupuytren’s disease, which would make inhibition of this pathway an interesting possible treatment target. Although systemic therapy influencing the Wnt pathway might not be a realistic solution, in view of its ubiquitous importance in cellular processes, local injections with Wnt-blocking agents as a preventive measure may be a possibility. However, our study does not address the question whether the activated Wnt pathway is the cause or the result of Dupuytren’s disease. In either case, inhibition of the Wnt pathway will have to be tested further in vitro and in vivo, before it would make a feasible treatment.

## Conclusion

In this study we report dysregulation of several Wnt-related genes and proteins, although not all genes found in the GWAS were changed. We found an increase in Wnt-target β-catenin in affected tissue, indicating activation of the canonical Wnt-signaling pathway. In addition, we found a substantial upregulation of Wnt7b, which might be a mediator of pro-fibrotic Wnt signaling in Dupuytren’s disease.

## Electronic supplementary material

Supplemental Figure 1. Characterization of Dupuytren’s disease tissue (cord, nodule) as compared to control tissue (unaffected transverse ligaments of the palmar aponeurosis). A) mRNA levels of *ACTA2*, *COL1A1* and *COL3A1*. * *P* < 0.05, *** *P* < 0.001 by Kruskal-Wallis test, followed by post-hoc Dunn’s Multiple Comparisons test. B) Representative pictures of α-smooth muscle actin staining in control, cord and nodule tissue of a Dupuytren’s disease patient (scale bar represents 50 μm). C) Representative pictures of collagen type I staining in control, cord and nodule tissue of a Dupuytren’s disease patient (scale bar represents 50 μm). D) Representative pictures of collagen type III staining in control, cord and nodule tissue of a Dupuytren’s disease patient (scale bar represents 50 μm). E) Quantification of stainings for α-smooth muscle actin, collagen type I and collagen type III, * *P* < 0.05 by Kruskal-Wallis test, followed by post-hoc Dunn’s Multiple Comparisons test. (GIF 449 kb)

High resolution image (TIFF 9068 kb)

## Abbreviations

GWAS: Genome-wide association study
TLPA: Transverse ligaments of the palmar aponeurosis
PBS: Phosphate buffered saline
q (RT)PCR: Quantitative (real-time) polymerase chain reaction
