# Supplementary figures and images for: Further evidence of the involvement of the Wnt signaling pathway in Dupuytren’s disease
Source: J Cell Commun Signal. 2015 Dec 3;10(1):33–40. doi: 10.1007/s12079-015-0312-8 (PMC4850140; doi:10.1007/s12079-015-0312-8)

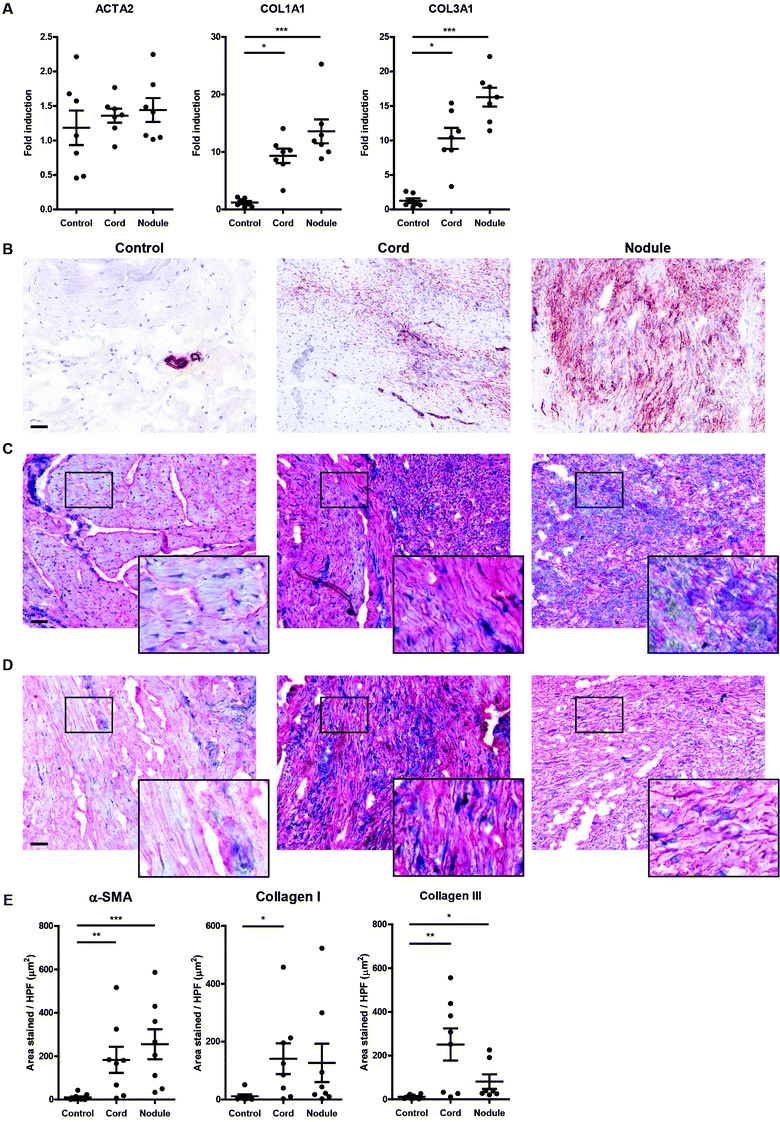

Supplement: Supplementary file 1 — Characterization of Dupuytren’s disease tissue (cord, nodule) as compared to control tissue (unaffected transverse ligaments of the palmar aponeurosis). A) mRNA levels of ACTA2, COL1A1 and COL3A1. * P < 0.05, *** P < 0.001 by Kruskal-Wallis test, followed by post-hoc Dunn’s Multiple Comparisons test. B) Representative pictures of α-smooth muscle actin staining in control, cord and nodule tissue of a Dupuytren’s disease patient (scale bar represents 50 μm). C) Representative pictures of collagen type I staining in control, cord and nodule tissue of a Dupuytren’s disease patient (scale bar represents 50 μm). D) Representative pictures of collagen type III staining in control, cord and nodule tissue of a Dupuytren’s disease patient (scale bar represents 50 μm). E) Quantification of stainings for α-smooth muscle actin, collagen type I and collagen type III, * P < 0.05 by Kruskal-Wallis test, followed by post-hoc Dunn’s Multiple Comparisons test. (GIF 449 kb) [file 12079_2015_312_MOESM1_ESM.gif]

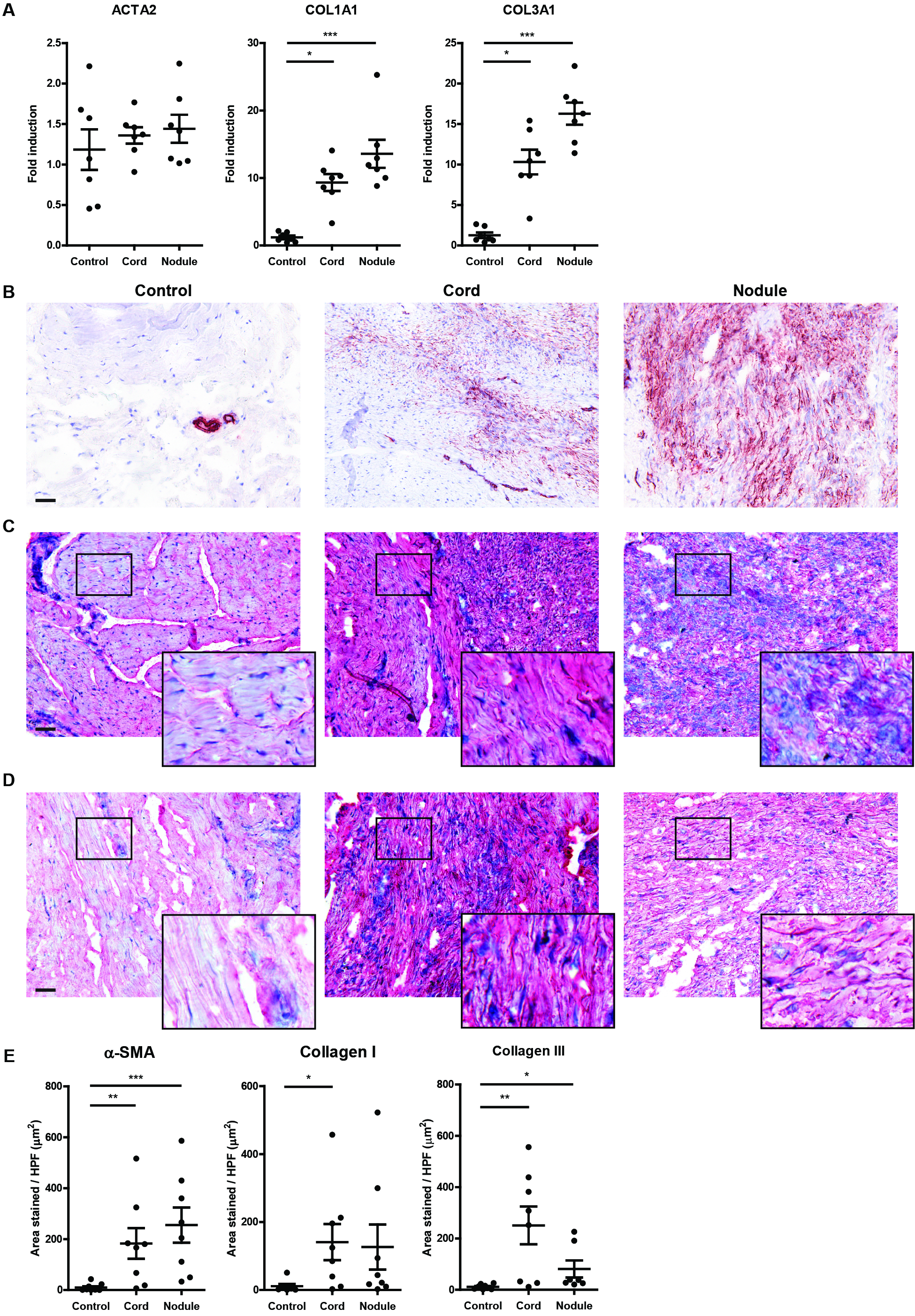

Supplement: Supplementary file 2 — High resolution image (TIFF 9068 kb) [file 12079_2015_312_MOESM2_ESM.tif]
